# Ubiquitin-specific peptidase 10, a deubiquitinating enzyme: Assessing its role in tumor prognosis and immune response

**DOI:** 10.3389/fonc.2022.990195

**Published:** 2022-09-28

**Authors:** Ziqi Ye, Jie Chen, Ping Huang, Zixue Xuan, Shuilian Zheng

**Affiliations:** ^1^ Department of Clinical Pharmacy, The First Affiliated Hospital, Zhejiang University School of Medicine, Hangzhou, China; ^2^ Zhejiang Provincial Key Laboratory for Drug Evaluation and Clinical Research, The First Affiliated Hospital, Zhejiang University School of Medicine, Hangzhou, China; ^3^ Department of Pharmacy, The Second Affiliated Hospital, Zhejiang University School of Medicine, Hangzhou, China; ^4^ Center for Clinical Pharmacy, Cancer Center, Department of Pharmacy, Zhejiang Provincial People’s Hospital (Affiliated People’s Hospital, Hangzhou Medical College), Hangzhou, China; ^5^ Key Laboratory of Endocrine Gland Diseases of Zhejiang Province, Zhejiang Provincial People’s Hospital (Affiliated People’s Hospital, Hangzhou Medical College), Hangzhou, China

**Keywords:** deubiquitination, epigenetic modification, tumorigenesis, immune response, USP10 inhibitors

## Abstract

Ubiquitin-specific peptidase 10 (USP10) is a member of the ubiquitin-specific protease family that removes the ubiquitin chain from ubiquitin-conjugated protein substrates. We performed a literature search to evaluate the structure and biological activity of USP10, summarize its role in tumorigenesis and tumor progression, and discuss how USP10 may act as a tumor suppressor or a tumor-promoting gene depending on its mechanism of action. Subsequently, we elaborated further on these results through bioinformatics analysis. We demonstrated that abnormal expression of USP10 is related to tumorigenesis in various types of cancer, including liver, lung, ovarian, breast, prostate, and gastric cancers and acute myeloid leukemia. Meanwhile, in certain cancers, increased USP10 expression is associated with tumor suppression. USP10 was downregulated in kidney renal clear cell carcinoma (KIRC) and associated with reduced overall survival in patients with KIRC. In contrast, USP10 upregulation was associated with poor prognosis in head and neck squamous cell carcinoma (HNSC). In addition, we elucidated the novel role of USP10 in the regulation of tumor immunity in KIRC and HNSC through bioinformatics analysis. We identified several signaling pathways to be significantly associated with USP10 expression, such as ferroptosis, PI3K/AKT/mTOR, TGF-β, and G2/M checkpoint. In summary, this review outlines the role of USP10 in various forms of cancer, discusses the relevance of USP10 inhibitors in anti-tumor therapies, and highlights the potential function of USP10 in regulating the immune responses of tumors.

## Introduction

Ubiquitination is an important epigenetic process that regulates transcription factors, histones, and chromatin states (1). A variety of biological processes are affected by ubiquitination in gene transcriptional regulation, including protein degradation (2), cell signaling (3), DNA damage repair (4), stress response (5), and cancer (6). Protein ubiquitination involves the addition of ubiquitin to target proteins through a multi-enzyme cascade, whereas deubiquitination is the removal of ubiquitin from proteins by deubiquitinating enzymes (DUBs) (7). As such, ubiquitination and deubiquitination constitute a dynamic equilibrium in cell biology. DUBs have become important drug targets for various diseases, such as tumors and neurodegenerative diseases (8, 9). Ubiquitin-specific peptidase 10 (USP10) is a member of the USP family of DUBs. It can remove conjugated ubiquitin from target proteins, such as autophagy-regulated gene Beclin1 (BECN1) (10), tumor protein p53 (TP53) (11), cystic fibrosis transmembrane conductance regulator (CFTR) (12), and sorting nexin 3 (SNX3) (13). It is also involved in a tumor necrosis factor receptor-associated factor (TRAF) family member-associated noncanonical nuclear factor-kappa B (NF-κB) activator (TANK)-dependent negative feedback response that attenuates NF-κB activation by deubiquitinating inhibitor of kappa polypeptide gene enhancer in B cells, kinase gamma (IKBKG) or tumor necrosis factor receptor-associated factor 6 (TRAF6) in response to interleukin-1-beta stimulation or DNA damage (14). Additionally, USP10 deubiquitinates and stabilizes T-box transcription factor protein (TBX21) (15). Moreover, it may affect the development of various cancers, including lung cancer (16), liver cancer (17), and acute myeloid leukemia (AML) (18). Interestingly, it also modulates immune-related signaling pathways *via* deubiquitination of immune-related genes, such as yes-associated protein 1 (YAP1) (19). The relationship between the abnormal expression of USP10 in various tumors and its regulation of immune pathways prompted us to explore the role of USP10 in tumor development and immunity. Here, we review the role of USP10 in tumors and regulation of immune responses and provide new insights into the USP family as potential targets for cancer therapy.

## Structure and biological roleof USP10

**Figure 1:**
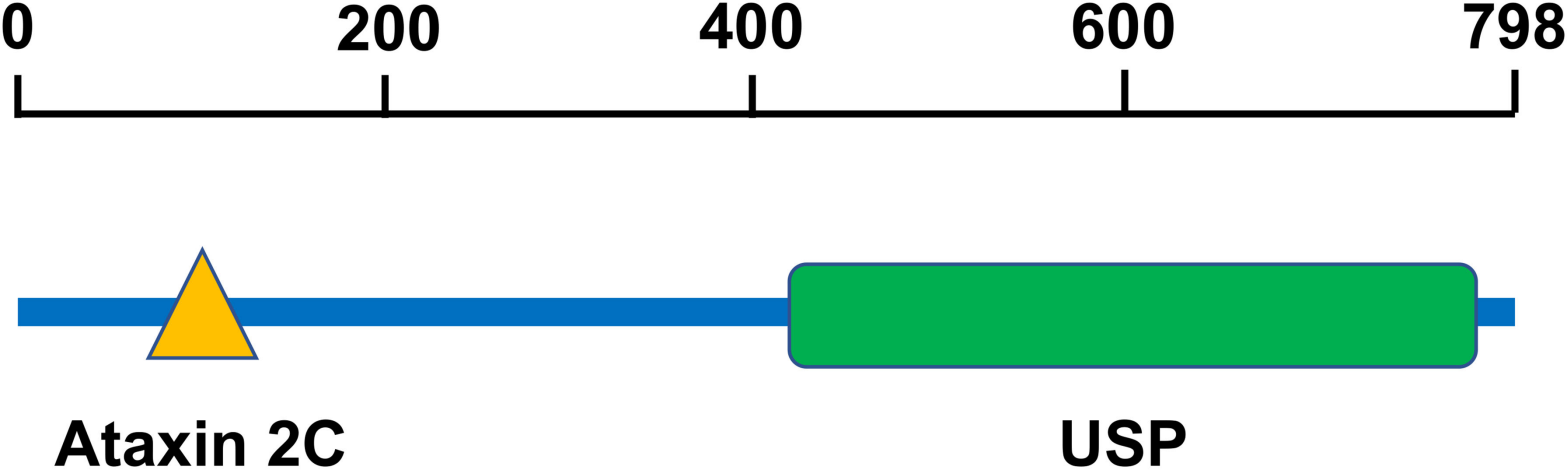
The structure of USP10. Human USP10 contains an Ataxin 2C domain and a USP domain. The Ataxin2C domain is approximately 250 residues long, and is located at the C-terminus of eukaryotic ataxin-2. The USP domain consists of a catalytic site, protein-protein interaction sites, and localization domains. USP10, Ubiquitin-specific peptidase 10.

USP10 is located on chromosome 16q24.1 (20) and consists of 798 amino acids (21). Human USP10 contains an Ataxin2C domain and a USP domain (**Figure 1**). The Ataxin2C domain is approximately 250 residues long, and is located at the C-terminus of eukaryotic ataxin-2. The function of ataxin-2 is unknown; however, the expansion of polyglutamine tracts may cause spinocerebellar ataxia type 2 (SCA2) (22). This expansion disrupts the normal morphology of the Golgi complex, leading to increased cell death (23). The USP domain consists of a catalytic site, protein-protein interaction sites, and localization domains. Since most USP domains require cleavage of the isopeptide bond between two ubiquitin molecules, a minimum of two ubiquitin-binding sites are required (24). The catalytic core of USP consists of six conserved boxes that are present in almost all USP domains. Boxes 1, 5, and 6 contain catalytic Cys, His, and Asp/Asn residues, respectively. Boxes 3 and 4 contain a Cys-X-X-Cys motif that functions as a zinc-binding motif. This zinc-binding motif facilitates the folding of the USP core, which facilitates the interaction between motifs separated by hundreds of residues (24).

USP10 has several molecular functions and characteristics. Firstly, USP10 has cysteine-type endopeptidase activity and can catalyze the hydrolysis of α-peptide bonds inside polypeptide chains through the sulfhydryl group of the cysteine residue in the active center (25). Secondly, USP10 may bind to members of one of the p53 protein families (26) and it may also bind to RNA molecules (27). USP10 also has a thiol-dependent iso-peptidase activity that cleaves ubiquitin from its conjugated target protein (25). Finally, USP10 may bind to transmembrane transporters, proteins, or protein complexes, facilitating the transfer of substances across membranes (28). Thus, USP10 is involved in a wide range of biological processes, including removal ubiquitin groups from proteins (26), negative regulation of the I-κB kinase/NF-κB signaling pathway and cellular responses to interleukin-1 and DNA damage stimuli (14), regulation of the cell cycle regulator phosphoprotein p53 in response to DNA damage (26), and in the regulation of autophagy (25). Furthermore, USP10 may also be involved in trans-lesion synthesis, which is the bypass of DNA lesions to overcome stalled replication at sites of DNA damage in the template strand. This is performed by a specialized DNA polymerase that allows DNA synthesis to continue by inserting a nucleotide at the lesion site (29). Thus, USP10 has a diverse array of relatively well characterized molecular and biological functions. However, its precise role in the development and progression of various forms of cancer remains to be elucidated.

## USP role in cancer

### USP10 in lung cancer

Lung cancer has the highest rates of cancer mortality worldwide, and multiple studies have investigated the role of USP10 in lung cancer. For example, Sun et al. reported that USP10 was significantly downregulated in lung cancer, and USP10 may directly interact with and stabilize the missing phosphatase and tensin homologue on chromosome 10 (PTEN) through deubiquitination, thereby acting as a tumor suppressor (30).

In non-small cell lung cancer (NSCLC), USP10 can deubiquitinate K63-linked polyubiquitination, thereby restoring the phosphatase activity of PTEN, reducing the secondary messenger phosphatidylinositol 3,4,5-triphosphate, which in turn attenuates AKT/mammalian target of rapamycin growth-promoting signaling, ultimately inhibiting NSCLC proliferation (31). Additionally, USP10 stabilizes and deubiquitinates MutS homolog 2 (MSH2), which is the core protein of the MutS homology family and is involved in DNA mismatch repair. These findings also suggest that USP10 downregulation leads to stabilization of the MSH2 protein, and thereby is implicated in the tumorigenesis of NSCLC (32, 33). Moreover, USP10 plays a role in regulating p53 ubiquitination and degradation. Zhao et al. reported that the half-life of p53 protein was significantly reduced in insulin-like growth factor 2 mRNA binding protein 3 (IGF2BP3)-overexpressing cells, and co-expression of IGF2BP3 with USP10 promoted the ubiquitination level of p53. Therefore, IGF2BP3 might attenuate the deubiquitination of p53 *via* USP10 (34).

Additionally, Ko et al. found that c-Myc could promote oncogene-induced senescence through the transcriptional activation of p14 alternate reading frame (p14ARF), followed by the activation of p53. Simultaneously, c-Myc increased the stability of p14ARF by inducing the transcription of USP10. Clinically, patients with NSCLC have significantly reduced overall survival (OS) due to disruption of the c-Myc-USP10-p14ARF axis (35). Hu et al. demonstrated that high levels of USP10 were associated with poor OS in TP53-mutant NSCLC but not in wild-type NSCLC. Likewise, USP10 knockout significantly reduced the growth of p53-mutated lung cancer xenografts and increased their sensitivity to cisplatin *in vivo*. Thus, USP10 might affect cisplatin resistance by deubiquitinating and thereby stabilizing the oncogenic protein histone deacetylase 6 (HDAC6) (36, 37). Another study showed that USP10 plays an important regulatory role in NSCLC *via* deubiquitinating and stabilizing histone deacetylase 7 (HDAC7), and that USP10 inhibition could significantly accelerate HDAC7 degradation and impair NSCLC proliferation and migration (38).

### USP10 in HCC

HCC has become one of the most common life-threatening cancers, due to its susceptibility to metastasis. Yuan et al. indicated that metastasis of advanced HCC might be closely related to the persistent activation of transforming growth factor-β (TGF-β) and Smad4. Yuan and colleagues found that USP10 deubiquitinates Smad4, maintains its protein expression level, and activates TGF-β signaling, thereby promoting HCC metastasis (39). Another study showed that the long non-coding RNA growth arrest associated lncRNA 1 (GASAL1), which may promote HCC progression, can upregulate USP10 expression by competitively binding to miR-193b-5p. Importantly, USP10 can also enhance HCC cell proliferation and migration by deubiquitinating proliferating cell nuclear antigen (PCNA) (40). Yes-associated protein (YAP) and its paralogs, transcriptional co-activators with a PDZ-binding motif (TAZ), play important roles in promoting HCC progression. Zhu et al. found that USP10 activated yes-associated protein/transcriptional coactivator with PDZ-binding motif (YAP/TAZ) activating gene and reduced proteolysis of the YAP/TAZ protein (17). In contrast to the above findings, Lu et al. found that USP10 could deubiquitinate and stabilize AMP-activated protein kinase α (AMPKα) and PTEN in HCC cells, leading to inhibition of mTOR Complex1 (mTORC1) and reduced AKT phosphorylation. Therefore, paradoxically, USP10 might also function as a tumor suppressor in HCC (41). USP10 may promote HCC proliferation on the one hand, yet on the other, it may function as a tumor suppressor. More research is required to clarify the precise role of USP10 in HCC.

### USP10 in AML

The 5-year OS rate for AML patients is only 20% (42). In particular, patients with FMS-like tyrosine kinase 3-internal tandem duplication (FLT3-ITD) mutations have a poor prognosis, as tumors with these mutations are associated with increased invasiveness and lethality (43). Furthermore, treatment with FLT3 kinase inhibitors has short-term efficacy due to development of drug resistance (44). Weisberg et al. revealed that USP10, a key enzyme for tumor growth in FLT3-ITD-positive AML, might inhibit FLT3 degradation through deubiquitination (18). The non-receptor tyrosine kinase spleen tyrosine kinase (SYK) is activated in FLT3-ITD-positive AML patients. This enzyme is critical for transformation and is associated with resistance to FLT3-targeting tyrosine kinase inhibitors (TKIs). Recent studies have shown that SYK and FLT3 are regulated by ubiquitination and deubiquitination (45–47). Interestingly, small-molecule inhibitors of USP10 have been found to inhibit the activated SYK-driven proliferation of leukemia cells and induce the degradation of SYK protein. USP10 can form a complex with FLT3-ITD and physically bind with SYK. USP10 stabilizes the expression of FLT3 and SYK by deubiquitination (48). Therefore, USP10 may be an effective drug target in kinase inhibitor-resistant AML.

### USP10 in ovarian cancer

Ovarian cancer (OC) is highly malignant and prevalent worldwide. Despite the high cure rates for early-stage OC, most patients are diagnosed with advanced tumors and the mortality rate is 75% (49). One study showed that reduced USP10 expression or reduced p14ARF/USP10 expression is an effective indicator of poor prognosis in OC patients (50). In addition, Li et al. proposed that GTPase-activating protein-binding protein 1 (G3BP1) could promote the proliferation and invasion of OC cells (51), and that USP10 knockdown could restore the proliferation and invasion of OC cells inhibited by G3BP1 knockdown. Thus, USP10 may act as a tumor suppressor in OC, however, more research is needed to elucidate its precise role.

### USP10 in prostate cancer

Prostate cancer (PCa) is a common malignancy among men, in which androgens and the androgen receptor (AR) play key roles in PCa tumorigenesis (52–54). Many patients are resistant to androgen deprivation therapy (ADT) or develop castration resistance (55, 56), therefore, there is an urgent need to find new therapeutic targets. USP10 stabilizes p53 by deubiquitination and thereby regulates AR-induced epigenetic signaling. GTPase-activating protein-binding protein 2 (G3BP2) is an androgen-responsive gene that can be stabilized by USP10-mediated deubiquitination. Therefore, USP10 may have an important oncogenic role in PCa by regulating the p53-G3BP2 complex and AR signaling (11). Recent studies have suggested that abnormal activation of the epidermal growth factor receptor (EGFR) might promote the progression of castration-resistant PCa by inhibition of androgen signaling (57, 58). Spautin-1, an inhibitor of USP10/13 (25), may inhibit EGFR-related signaling pathways, inducing activation of the MKK4/JNK/Bax axis and inactivation of the MEK1/2/ERK/cyclin D1 axis, leading to significant inhibition of PCa proliferation (59).

### USP10 in breast cancer

Breast cancer (BC) is one of the most common cancers, accounting for 30% of all cancer cases in women (60, 61). Yang et al. identified a novel circRNA, circWSB1, which is highly expressed in BC tissues and strongly associated with poor prognosis in patients with BC. Upon direct binding to USP10, circ WSB1 can reduce USP10-mediated p53 stability, resulting in p53 degradation and promoting BC development (62). USP10 may also contribute to BC progression through topoisomerase IIα (TOP2α), which is essential for chromosome condensation, segregation, and genome integrity (63). RING finger protein 168 (RNF168), an E3 ligase, interacts with TOP2α and mediates its ubiquitination. RNF168 deficiency impairs TOP2α activity and promotes mitotic abnormalities and chromosome segregation defects. RNF168 deficiency in human breast cancer cell lines leads to drug resistance, including resistance to the TOP2 inhibitor, etoposide. USP10 may deubiquitinate TOP2α and inhibit chromatin binding. Therefore, interactions between USP10, RNF168, and TOP2α may play a key role in the development of tumor resistance to TOP2 inhibitors (64).

### USP10 in gastric cancer

Gastric cancer (GC) is highly malignant, with 700,000 GC-related deaths occurring annually worldwide (65). Zeng et al. demonstrated that the expression level of USP10 in GC tissues and cell lines was lower than that in non-cancerous mucosal tissues and gastric epithelial immortalized cell lines. Moreover, the expression level of USP10 was negatively correlated with invasion and lymph node metastasis in GC. Low USP10 expression was significantly associated with poor prognosis in GC patients. Furthermore, multivariate analysis indicated that USP10 was an independent prognostic factor for OS in patients with GC (20).

### USP10 in small intestinal adenocarcinoma

Small intestinal adenocarcinoma is rare, accounting for only 2% of gastrointestinal malignancies (66). p14ARF, encoded by a reading frame within the cyclin-dependent kinase inhibitor p16/the p53 regulator p14 (INK4a/ARF) locus on chromosome 9p21 (67), can inhibit the E3 ubiquitin protein ligase mouse double minute 2 (MDM2)-mediated degradation of TP53, thereby increasing TP53 stability and leading to cell cycle arrest and apoptosis (68). Song et al. revealed that USP10 deletion is associated with advanced tumor-related phenotypes, and that co-deletion of USP10 and p14ARF yields poor outcomes in small intestinal adenocarcinoma, indicating that USP10 and p14ARF may be involved in small intestinal adenocarcinoma (69).

### USP10 in colorectal cancer

Li et al. reported that NACHT, LRR, and PYD domain-containing protein 7 (NLRP7) plays a key oncogenic role in the proliferation and metastasis of colorectal cancer (CRC) and is associated with poor prognosis. USP10-mediated deubiquitination stabilized NLRP7 protein expression and induced polarization of tumor-promoting M2-like macrophages through NF-κB pathway-mediated monocyte chemoattractant protein-1 (MCP-1) secretion (70). In addition, the tumor suppressor sirtuin 6 (SIRT6) is significantly inversely correlated with tumorigenesis (71), and USP10 can inhibit the transcriptional activity of the c-Myc by increasing the protein stability of SIRT6 and p53, ultimately inhibiting the progression of CRC (72). Kim et al. also noted that USP10 expression was absent in 18.6% of CRC tissue and was significantly associated with distant metastasis and lymph vascular invasion. Likewise, USP10 deletion was associated with shorter OS, disease-free survival, and was an independent prognostic factor in CRC patients (73). In contrast, another study showed that USP10 promoted CRC cell proliferation by deubiquitinating and stabilizing the oncogenic factor musashi 2 (MSI2) (74).

### USP10 in pancreatic cancer

Pancreatic cancer is an extremely lethal malignancy with a high mortality rate worldwide (75). The role of USP10 in pancreatic cancer remains controversial. For example, Liu et al. reported that USP10 promoted Cysteine-Rich 61 (Cyr61) expression by inhibiting YAP1 ubiquitination and degradation, thereby favoring immune escape and promoting the proliferation and metastasis of pancreatic cancer (19). However, another study suggested that miR-191 promotes pancreatic cancer cell proliferation by inhibiting USP10 expression (76). Furthermore, it has been reported that miR-103 may also downregulate USP10 in pancreatic cancer cell lines and tissues. Upregulation of miR-103 expression is associated with lymph node metastasis, advanced TNM stage, and poor prognosis (77). Thus, the specific regulatory role of USP10 in pancreatic cancer requires further investigation.

### USP10 in other tumors

Neurotrophin receptor-interacting MAGE homologue (NRAGE) is generally considered to be a tumor suppressor, yet Yang et al. found that NRAGE significantly promoted esophageal carcinoma, mainly *via* upregulation of PCNA. Knockdown of NRAGE promoted PCNA K48-linked polyubiquitination, leading to proteasome-dependent degradation of PCNA and cancer proliferation inhibition, and USP10 is a key regulator in this process (78).

In chronic myeloid leukemia (CML), constitutive activation of the tyrosine kinase Bcr-Abl is a major cause of disease development and progression (79). However, acquired resistance to Bcr-Abl-targeted TKIs severely affects the prognosis of patients with advanced CML. Liao et al. reported that as a co-regulator of Bcr-Abl, S-phase kinase-associated protein 2 (SKP2) mediates activation of its K63 linkages. USP10 can further enhance Bcr-Abl activation by promoting the deubiquitination of SKP2 in CML cells, and stabilizing its protein expression. This study demonstrated that targeting the USP10/SKP2/Bcr-Abl axis could reverse imatinib resistance in CML patients (80).

In glioblastoma (GBM), one study showed that Cyclin D1 (CCND1) is involved in cell cycle control and promotes tumor progression (81). In this study, Sun et al. found that USP10 may interact with CCND1 and prevent its K48-linked polyubiquitination, increasing CCND1 stability. Overall, the USP10/CCND1 axis is expected to be an effective target for the treatment of GBM (82).

In thyroid cancer, Cui et al. reported that 3-deazaneplanocin A (DZNep) may cause the accumulation of p53 protein by upregulating USP10 expression, thereby activating the p53 pathway and ultimately inhibiting the growth of TP53 wild-type thyroid cancer cells (83).

The role of USP10 in different tumors, as discussed above, indicate that the deubiquitination substrates of USP10 are diverse and that USP10 targets different signaling pathways (**Table 1**). Additionally, we found that USP10 acts as an oncogene in some cancers but not in others, due to the different functions of genes deubiquitinated by USP10. For example, if the gene deubiquitinated by USP10 is an oncogene, it may lead to tumor progression, and if the gene deubiquitinated by USP10 is a tumor suppressor gene, it may lead to tumor suppression. In short, USP10 cannot be simply defined as a tumor suppressor gene or an oncogene, and its function may be directly related to the function of its deubiquitinated gene, rather than USP10 itself having oncogenic or tumor suppressor effects. In conclusion, USP10 plays a diverse set of roles in either promoting or inhibiting the progression of various of cancers. Clinically, it may serve as a new tumor biomarker and potential therapeutic target, however, its precise role would be dependent on the cancer type.

**Table 1:** The deubiquitination substrates and downstream signaling pathways of USP10 in different cancer types.

Cancer types	Deubiquitination substrates	Downstream signaling pathways
Lung cancer	PTEN, MSH2, p53, p14ARF, HDAC,	AKT/mTOR
Hepatocellular carcinoma	Smad4, GASAL1, PCNA, YAP/TAZ, AMPKα, PTEN	TGF-β, AKT/mTOR
Acute myeloid leukemia	FLT3, SYK	/
Ovarian cancer	p14ARF, G3BP1	/
Prostate cancer	P53, G3BP2, EGFR	Androgen receptor signaling, MKK4/JNK/Bax, MEK1/2/ERK/cyclinD1
Breast cancer	p53, Top2α	/
Gastric carcinoma	p53	/
Small intestinal adenocarcinoma	Synergistic with p14ARF, TP53	/
Colorectal cancer	NLRP7, SIRT6, p53, MSI2	NF-κB
Pancreatic cancer	YAP1	/
Esophageal carcinoma	PCNA	/
Chronic myeloid leukemia	SKP2,	Bcr-Abl
Glioblastoma	CCND1	/
Thyroid cancer	p53	

USP10, Ubiquitin-specific peptidase 10; PTEN, phosphatase and tensin homologue on chromosome 10; MSH2, MutS homolog 2; p14ARF, p14 alternate reading frame; HDAC, histone deacetylase; GASAL1, growth arrest associated lncRNA 1; PCNA, proliferating cell nuclear antigen; YAP/TAZ, yes-associated protein/transcriptional coactivator with PDZ-binding motif; AMPKα, AMP-activated protein kinase α; FLT3, FMS-like tyrosine kinase 3; SYK, spleen tyrosine kinase; G3BP1, GTPase-activating protein-binding protein 1; G3BP2, GTPase-activating protein-binding protein 2; EGFR, epidermal growth factor receptor; TOP2α, topoisomerase IIα; TP53, tumor protein p53; NLRP7, NACHT, LRR, and PYD domain-containing protein 7; SIRT6, sirtuin 6; MSI2, musashi 2; YAP1, yes-associated protein 1; SKP2, S-phase kinase-associated protein 2; CCND1, Cyclin D1.

## USP10 inhibitors and other modulators of USP10 activity

The ubiquitin-binding site in USP10 functions as a thiol-dependent isopeptidase, which separates ubiquitin from target proteins, and thereby mediates their stability (84). In this context, USP10 inhibitors that target the binding site might effectively interfere with its oncogenic effects. USP10 expression levels differ widely across various tumors and are significantly correlated with the prognosis of these tumors. Thus, USP10 inhibitors may be clinically useful in cancer settings where overexpression of USP10 is associated with tumor progression or in settings where USP10 serves as an oncogene. To date, only a few USP10 inhibitors have been identified, P22077, HBX19818, and spautin-1 (85). P22077 and HBX19818 were first identified as irreversible USP7 inhibitors. However, one study showed that P22077 and HBX19818 might also inhibit the proliferation of FLT3-ITD-positive tumor cells by inhibiting the deubiquitinase activity of USP10 (18). Spautin-1, a small-molecule inhibitor, has been shown to inhibit the deubiquitinase activities of both USP10 and USP13, leading to increased ubiquitination and degradation of Beclin1 in the Vps34 complex, and an eventual decrease in the level of autophagy in cancer cell lines (25). In addition, spautin-1 inhibition of USP10 significantly attenuates the migration of HCC cells (39). Interestingly, spautin-1 also inhibits the proliferation of prostate cancer, NSCLC, ovarian cancer, and melanoma cells in a USP10-independent manner (86). Spautin-A41 (an analog of spautin-1), was recently found to be more effective than spautin-1 in inhibiting autophagy and inducing microsomal stability (87). However, the mechanism of action of spautin-1 and spautin-A41 in cancer patients still needs to be confirmed. Recently, compound library screening revealed that Wu-5, a novel USP10 inhibitor, enhanced the anti-AML effect of crenolanib and reversed FLT3 inhibitor resistance. Mechanistically, Wu-5 inhibited the proliferation of MV4-11 cells mainly by inhibiting the activity of USP10 and subsequently reducing the expression of the downstream gene AMPKα (88).

There are also several USP10 modulators that indirectly influence USP10 activity. For example, DZNep stabilizes p53 by upregulating USP10 to reduce ubiquitin binding in wild-type GC cells, thereby activating p53 and inhibiting the proliferation of GC cells (89). Quercetin (C15H10O7) is a pentahydroxy flavonoid widely present in vegetables and fruits, and it has been shown that quercetin could reduce USP10 expression, resulting in the downregulation of T-bet expression levels (15). Quercetin inhibits the proliferation of several cancer cells, including colon cancer, breast cancer, and lung cancer (90). However, the mechanism of how quercetin inhibits USP10 remains unclear. Another potential USP10 modulator, Cai’s Neiyi prescription (CNYP), can alleviate inflammation by inhibiting USP10 and promoting apoptosis of endometrial stromal cells, suggesting that CNYP may be an inhibitor of USP10 (91). Unlike other USP10 inhibitors, ubiquitin variant.10.1 (UbV.10.1) is a mixture of peptides and proteins with a high affinity for USP10. UbV.10.1 overexpression can promote the degradation of p53 by inhibiting USP10 (92). However, the antitumor effects of both CNYP and UbV.10.1 have yet to be explored.

No USP10 inhibitors have been considered in clinical trials for cancer treatment. This is likely owing to the low selectivity of most USP10 inhibitors. As such, technologies such as polymer chemistry and selective structural modification may offer strategies to improve both the anti-tumor effects of USP10 inhibitors and their selectivity for tumor cells. Furthermore, it would be necessary to identify suitable patients for USP10 inhibitor treatment based on their cancer type. Considering the immunoregulatory role of USP10, combining USP10 inhibitors with immunotherapy may be a promising future research avenue.

## Bioinformatics analysis

### Abnormal USP10 expression in tumors

**Figure 2:**
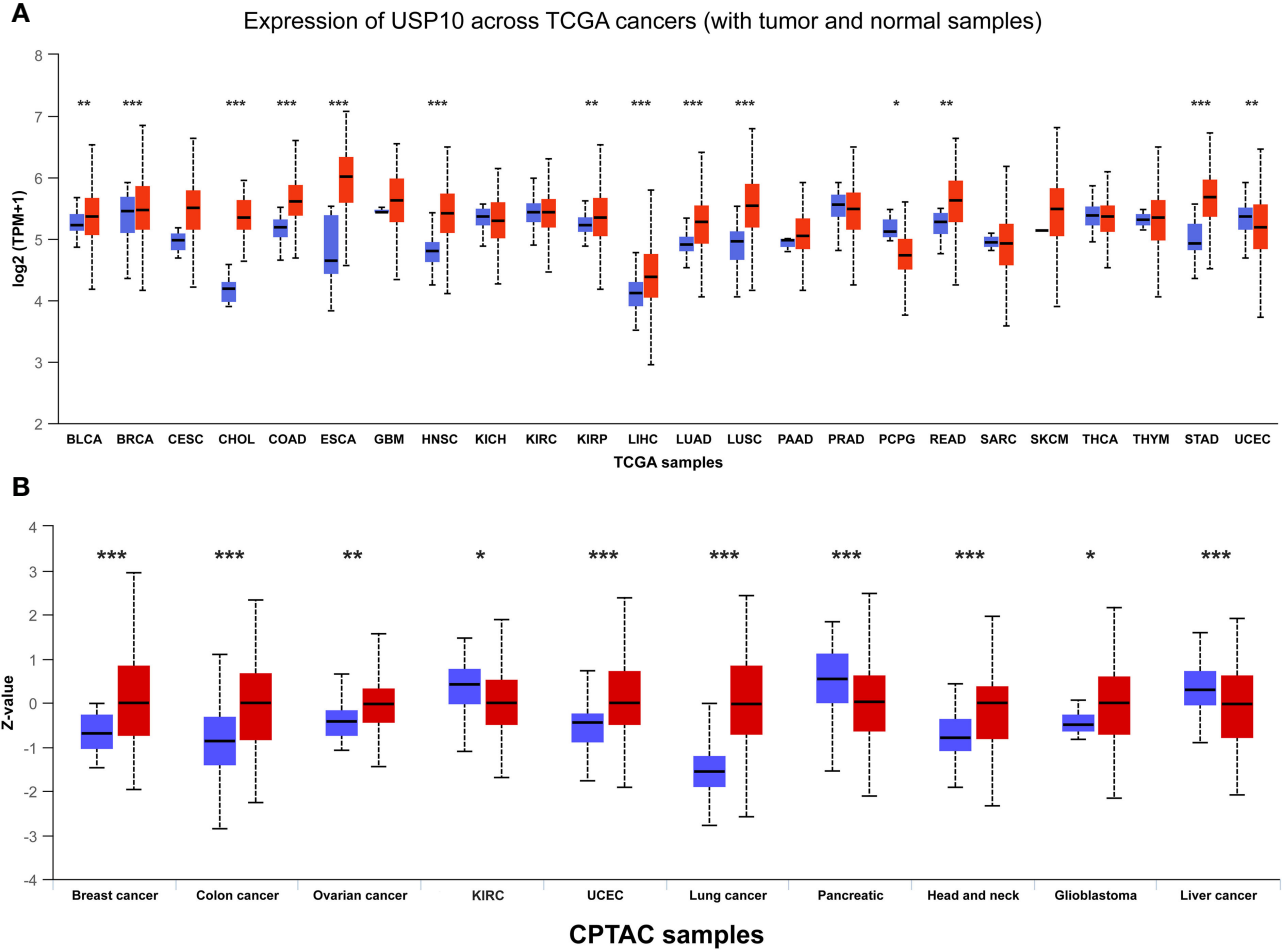
The gene and protein expressions of USP10 in pan-cancers based on TCGA and CPTAC databases. **(A)** The gene expressions of USP10 in pan-cancers based on TCGA database; **(B)** The protein expressions of USP10 in pan-cancers based on CPTAC database. Red color: tumor tissues; Blue color: normal tissues. USP10, Ubiquitin-specific peptidase 10; TCGA, The Cancer Genome Atlas; CPTAC, Clinical Proteomic Tumor Analysis Consortium. *P < 0.05; **P < 0.01; ***P < 0.001.

After discussing the literature and knowledge gaps above, we found that the role of USP10 in cancer is conflicting. Therefore, to further examine the expression of USP10 in cancer, we compared the differences in USP10 expression between tumor tissues and adjacent normal tissues by analyzing gene expression data of USP10 in different human tumor samples from The Cancer Genome Atlas (TCGA) database. The University of ALabama at Birmingham CANcer data analysis Portal (UALCAN) (93) showed that USP10 gene expression was significantly upregulated in 12 types of tumor samples: breast invasive carcinoma, bladder urothelial carcinoma, cholangiocarcinoma, esophageal carcinoma, colon adenocarcinoma, kidney renal papillary cell carcinoma, head and neck squamous cell carcinoma (HNSC), lung adenocarcinoma, liver hepatocellular carcinoma, lung squamous cell carcinoma, stomach adenocarcinoma, and rectum adenocarcinoma. Conversely, significant downregulation of USP10 gene expression was observed in two types of tumor samples: pheochromocytoma and paraganglioma (PCPG) and uterine corpus endometrial carcinoma (UCEC) (**Figure 2A**). Based on data from the International Cancer Proteogenome Consortium (ICPC) and the Clinical Proteomic Tumor Analysis Consortium (CPTAC), we analyzed differences in USP10 protein expression between tumor tissues and adjacent normal tissues (94). USP10 protein expression was significantly upregulated in seven types of tumor samples: breast cancer, colon cancer, ovarian cancer, UCEC, lung cancer, HNSC, and glioblastoma. However, significant downregulation of USP10 protein expression was observed in three types of tumor samples: kidney renal clear cell carcinoma (KIRC), pancreatic cancer, and liver cancer (**Figure 2B**). Interestingly, USP10 was highly expressed in lung cancer samples in the TCGA and CPTAC databases (**Figure 2**). These results appear to be inconsistent with those reported in the literature. We speculated that it may be due to tumor heterogeneity or the insufficient sample size. Therefore, whether USP10 expression is increased or decreased in lung cancer and whether USP10 plays a role in promoting or suppressing lung cancer require further investigation.

**Figure 3:**
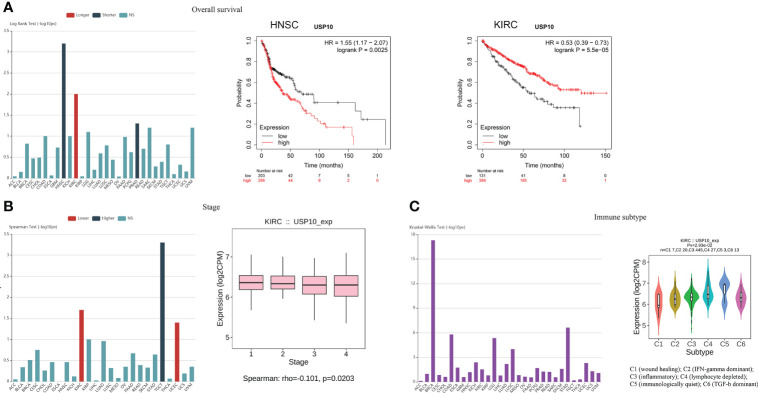
Analysis of the TISIDB and Kaplan-Meier plotter website indicated the role of USP10 in tumors and immune subtype. **(A)** HNSC patients with increased USP10 expression had lowered OS, however, high levels of USP10 were associated with prolonged OS in KIRC patients. **(B)** The correlation between USP10 expression and tumor stage in KIRC patients. **(C)** USP10 was correlated with the immune subtype in KIRC. NS, not significant. USP10, Ubiquitin-specific peptidase 10; HNSC, head and neck squamous cell carcinoma; OS, overall survival; KIRC, kidney renal clear cell carcinoma.

In addition, we investigated whether there was a correlation between USP10 expression and OS or tumor stage. Data collected from the TISIDB website (an integrated repository portal for tumor-immune system interactions) showed that HNSC and prostate adenocarcinoma (PRAD) patients with increased USP10 expression had shorter OS, suggesting that USP10 plays an oncogenic role in HNSC and PRAD (95). Further validation analysis (https://kmplot.com/analysis/) revealed that low levels of USP10 expression was significantly associated with prolonged OS in HNSC patients (**Figure 3A**). However, USP10 was downregulated in KIRC, and high levels of USP10 was associated with prolonged OS in KIRC patients, suggesting that USP10 may have a tumor suppressor role in KIRC (**Figure 3A**). In a pan-cancer analysis, we found that high USP10 expression was associated with early tumor stage in KIRC and UCEC patients. However, in testicular germ cell tumor (TGCT) patients, high USP10 expression predicted later tumor stage (**Figure 3B**). Consistent with these findings, another study also has demonstrated the close relationship between USP10 expression and the progression and prognosis of various cancers, including lung cancer, hepatocellular carcinoma (HCC), ovarian cancer, prostate cancer, and AML (96).

### USP10 regulates the immune response in tumors

Deubiquitination is a common post-translational modification that modulates multiple cellular functions, including protein stability. Meanwhile, deubiquitination also affects multiple signal transduction pathways, including some immunomodulatory pathways (97). For example, USP10 promotes the deubiquitination of tumor necrosis factor receptor-associated factor 6 (TRAF6) and inhibits NF-κB and the interleukin-1 receptor/Toll-like receptor (IL-1R/TLR) activation (14). TLRs can promote inflammation and immune response in tumors, suggesting a potential role for USP10 in the regulation of immune pathways. Growing evidence suggests that USP10 may be involved in the infiltration of various immune cells in tumors, potentially regulating the infiltration levels of specific immune cells (98); however, its precise role in tumor immune response remains unclear. We further analyzed data from the TISIDB (http://cis.hku.hk/TISIDB/index.php) to evaluate the correlation between USP10 and immunostimulatory molecules, immunosuppressive molecules, major histocompatibility complex (MHC) molecules, chemokines, and MHC receptors, to better understand the immune function of USP10 in cancer regulation.

**Figure 4:**
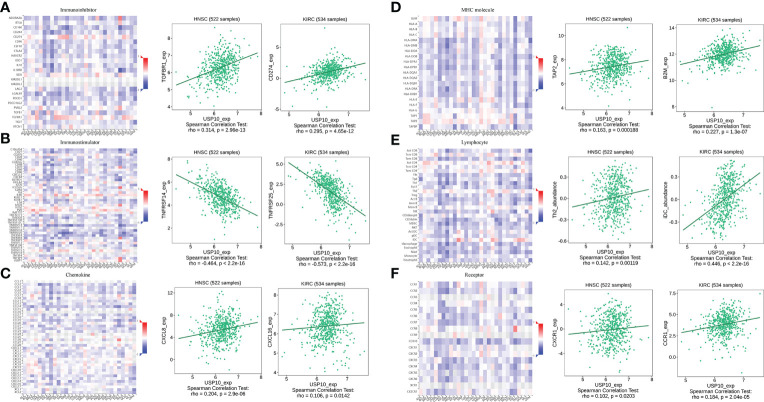
USP10 was positively correlated with immunosuppressive molecules **(A)**, immunostimulatory molecules **(B)**, chemokines **(C)**, MHC molecules **(D)**, lymphocytes **(E)**, and MHC receptors **(F)**. USP10, Ubiquitin-specific peptidase 10; MHC, major histocompatibility complex.

**Figure 5:**
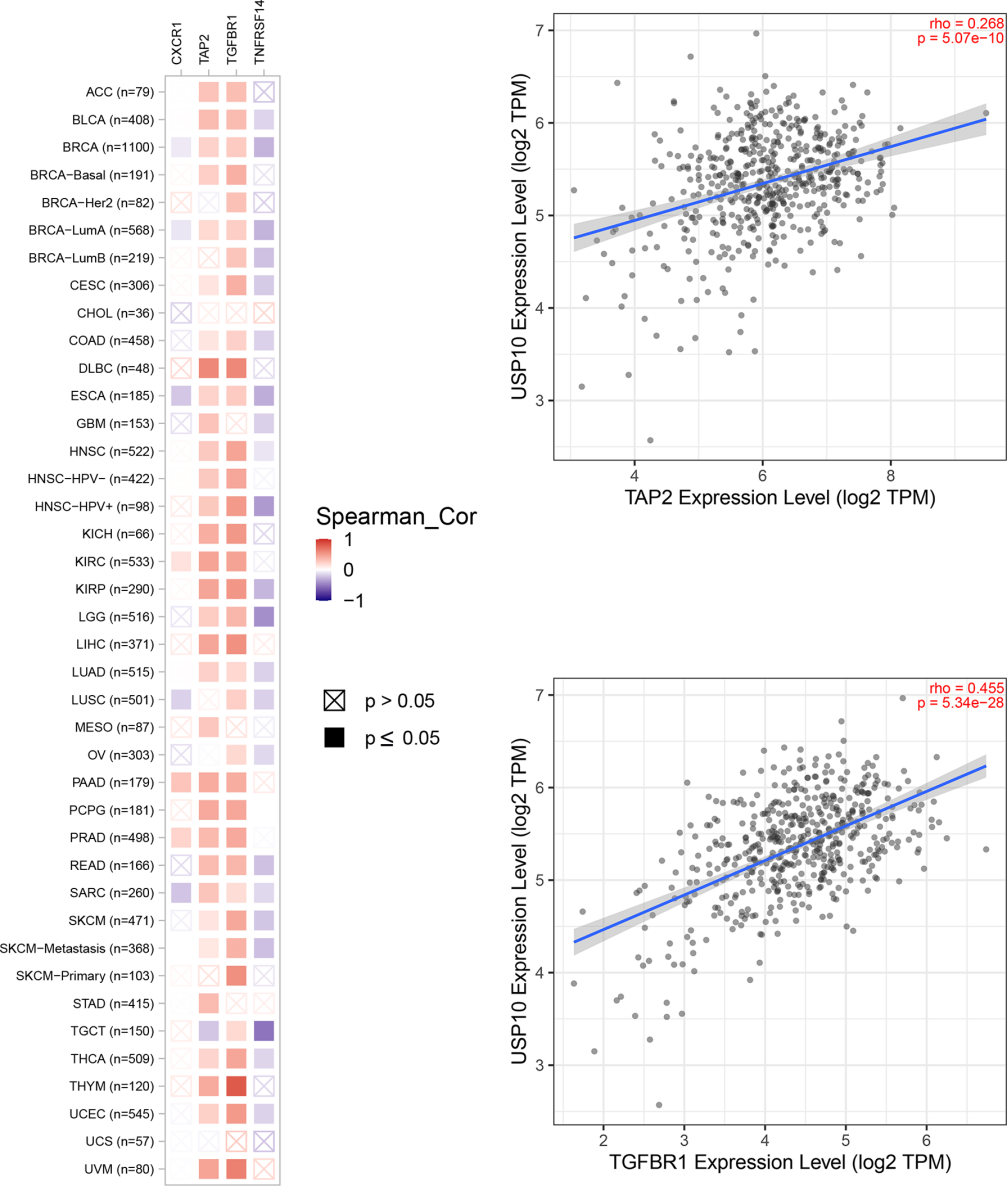
USP10 was positively correlated with TAP2 and TGFBR1 in HNSC.

**Figure 6:**
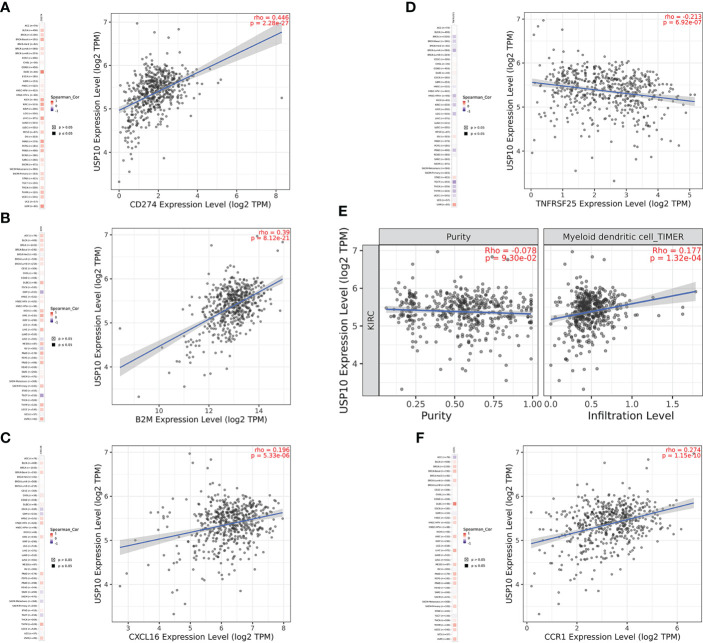
USP10 was positively correlated with CD274 **(A)**, B2M **(B)**, CXCL16 **(C)**, myeloid dendritic cell **(E)**, and CCR1 **(F)**, but negatively correlated with TNFRSF25 **(D)** in KIRC. USP10, Ubiquitin-specific peptidase 10; TAP2, transporter associated with antigen processing 2; TGFBR1, transforming growth factor beta receptor 1; HNSC, head and neck squamous cell carcinoma; B2M, β2-microglobulin; CXCL16, C-X-C motif ligand 16; CCR1, CC Chemokine receptor 1; TNFRSF25, TNF receptor superfamily 25; KIRC, kidney renal clear cell carcinoma.

In HNSC, USP10 was positively correlated with the immunosuppressive molecules transforming growth factor beta receptor 1 (TGFBR1), the immunostimulator tumor necrosis factor receptor superfamily member 14 (TNFRSF14), the chemokine C-X-C motif ligand 8 (CXCL8), the MHC molecule transporter associated with antigen processing 2 (TAP2), lymphocyte T helper type (Th2), and the MHC receptor C-X-C chemokine receptor 1 (CXCR1) (P<0.05, **Figure 4**). In KIRC, USP10 was correlated with the immune subtype (P=0.029) (**Figure 3C**). Furthermore, USP10 was positively correlated with the immunosuppressive molecules CD274, immunostimulator TNF receptor superfamily 25 (TNFRSF25), chemokine C-X-C motif ligand 16 (CXCL16), β2-microglobulin (B2M), lymphocyte immature dendritic cells (iDC), and MHC receptor CC Chemokine receptor 1 (CCR1) (P<0.05, **Figure 4**). Analysis of the TIMER database (http://timer.cistrome.org/) verified the correlation of USP10 with these immune markers (**Figures 5**, **6**). CD274 plays a key role in the induction and maintenance of self-immune tolerance. As a ligand for the inhibitory receptor programmed cell death 1 (PDCD1)/CD279, CD274 modulates the activation threshold of T cells and limits T cell effector responses (99). Tumors utilize PDCD1/CD279-mediated inhibitory pathways to attenuate antitumor immunity and promote tumor survival (100). CXCL16 is a chemotactic agent for immunosuppressive regulatory T cells (Treg), which may be involved in Treg recruitment and tumorigenic functions (101). B2M is an important subunit of MHC class I and plays important biological functions in tumorigenesis and immune control. There is increasing evidence that alterations in B2M genes and proteins contribute to poor responses to cancer immunotherapy by inhibiting antigen presentation (102). CCR1 is a receptor for C-C type chemokines. It may play a role in Treg and tumor immunosuppression (103). TNFRSF25 is a member of the TNF receptor superfamily (TNFRSF) that binds to the TNF-like protein TL1A. Recent studies have demonstrated a role for TNFRSF25 in regulating CD4+ T cell responses. Additionally, TNFRSF25 signaling in CD8+ T cells positively affects the proliferation of CD8+ T cells and their differentiation into CTLs (104). The immature DC phenotype can reduce dendritic cell function, reduce antigen presentation, and affect T cell effector function (105). The above results suggest that USP10 may play an immunosuppressive function by promoting the expression of some immunosuppressive cytokines or by inhibiting the expression of some immune-activating cytokines. However, the specific immune regulation mechanism of USP10 remains to be fully elucidated.

**Figure 7:**
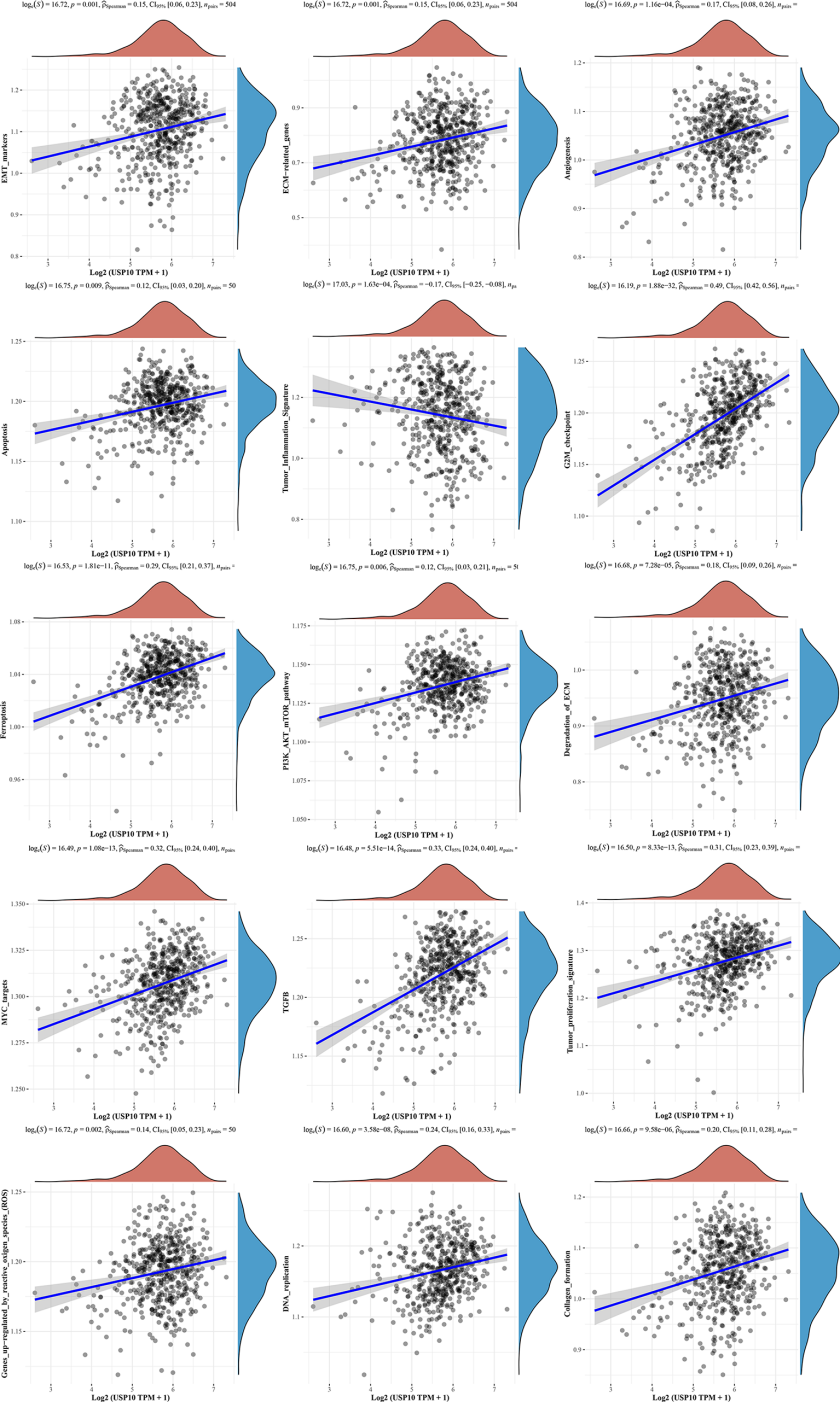
The signaling pathways significantly related to USP10 in HNSC included EMT, ECM, angiogenesis, apoptosis, tumor inflammation, G2/M checkpoint, ferroptosis, PI3K/AKT/mTOR, MYC, TGF-β, tumor proliferation, reaction oxygen species, DNA replication, and collagen formation. USP10, Ubiquitin-specific peptidase 10; HNSC, head and neck squamous cell carcinoma; EMT, Epithelial-mesenchymal transition; ECM, extracellular matrix.

**Figure 8:**
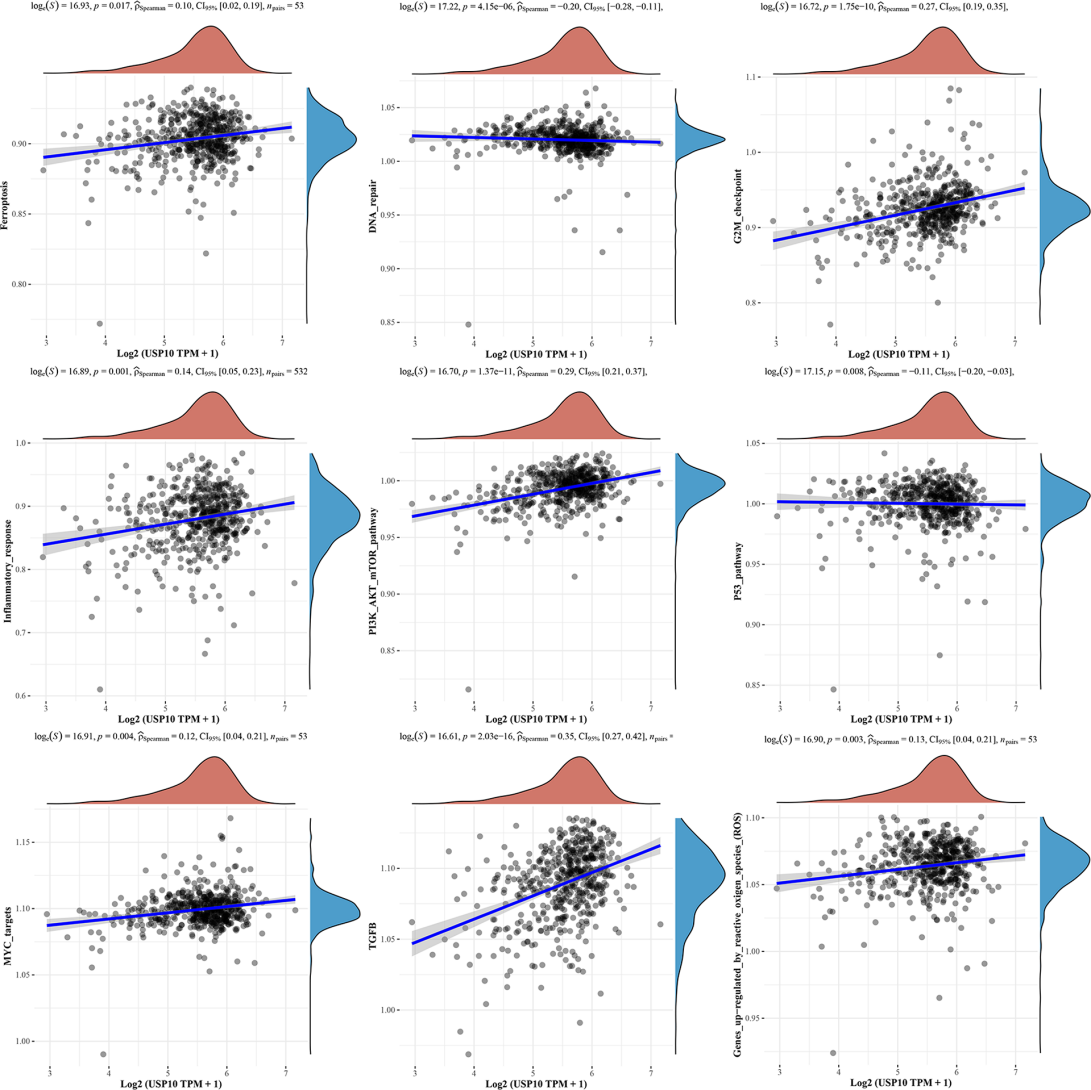
The signaling pathways significantly related to USP10 in KIRC included ferroptosis, DNA repair, G2/M checkpoint, inflammatory response, PI3K/AKT/mTOR, p53, c-Myc, TGF-β, and ROS. USP10, Ubiquitin-specific peptidase 10; KIRC, kidney renal clear cell carcinoma; ROS, reactive oxygen species.

We also analyzed the correlations between USP10 and pathway scores by Spearman’s correlation. In HNSC, USP10 was found to be correlated with Epithelial-mesenchymal transition (EMT), extracellular matrix (ECM), angiogenesis, apoptosis, tumor inflammation, G2/M checkpoint, ferroptosis, PI3K/AKT/mTOR, MYC, TGF-β (**Figure 7**). In KIRC, we found that the signaling pathways significantly related to USP10 included ferroptosis, DNA repair, G2/M checkpoint, inflammatory response, PI3K/AKT/mTOR, p53, c-Myc, TGF-β, and reactive oxygen species (ROS) (**Figure 8**). In summary, we suggest that USP10 maybe a novel target for cancer immunotherapy; however, the specific mechanisms of USP10 involvement in cancer immunity remain unclear and further research is required.

## Discussion

USP10 acts as a tumor suppressor or tumor-promoting gene depending on the target genes modified by deubiquitination. In this paper, we have discussed the biological function of USP10 and its key role in tumor progression and immune response. We have demonstrated that USP10 is a potential drug target for immunotherapy in tumors. In addition, we have summarized the current knowledge regarding USP10 inhibitors. However, it remains unclear how USP10 regulates immunity and whether USP10-mediated deubiquitination may affect tumor response to immunotherapy. Therefore, studying the role and mechanism of USP10 immune regulation will provide a theoretical basis for the clinical application of USP10-oriented therapies in the future.

## Author contributions

ZY and ZX designed this study, JC, ZY, and SZ edited the manuscript and contributed to the systematic evaluation of articles and literature. ZX and PH participated in the writing and revision of the manuscript, and all authors approved the final version of the article and agreed to be responsible for all aspects of the work.

## Funding

This study was supported by the Joint Fund of Zhejiang Provincial Natural Science Foundation (Grant No. LYY21H310005), Natural Science Foundation of Zhejiang Province (Grant Number LYY21H310008), Zhejiang Traditional Chinese Medicine Science and Technology Plan (Grant Number 2022ZQ014).

## Conflict of interest

The authors declare that the research was conducted in the absence of any commercial or financial relationships that could be construed as a potential conflict of interest.

## Publisher’s note

All claims expressed in this article are solely those of the authors and do not necessarily represent those of their affiliated organizations, or those of the publisher, the editors and the reviewers. Any product that may be evaluated in this article, or claim that may be made by its manufacturer, is not guaranteed or endorsed by the publisher.
